# The Modular Organization of Domain Structures: Insights into Protein–Protein Binding

**DOI:** 10.1371/journal.pcbi.0030239

**Published:** 2007-12-07

**Authors:** Antonio del Sol, Pablo Carbonell

**Affiliations:** Bioinformatics Research Unit, Research and Development Division, Fujirebio, Tokyo, Japan; Weizmann Institute of Science, Israel

## Abstract

Domains are the building blocks of proteins and play a crucial role in protein–protein interactions. Here, we propose a new approach for the analysis and prediction of domain–domain interfaces. Our method, which relies on the representation of domains as residue-interacting networks, finds an optimal decomposition of domain structures into modules. The resulting modules comprise highly cooperative residues, which exhibit few connections with other modules. We found that non-overlapping binding sites in a domain, involved in different domain–domain interactions, are generally contained in different modules. This observation indicates that our modular decomposition is able to separate protein domains into regions with specialized functions. Our results show that modules with high modularity values identify binding site regions, demonstrating the predictive character of modularity. Furthermore, the combination of modularity with other characteristics, such as sequence conservation or surface patches, was found to improve our predictions. In an attempt to give a physical interpretation to the modular architecture of domains, we analyzed in detail six examples of protein domains with available experimental binding data. The modular configuration of the TEM1-β-lactamase binding site illustrates the energetic independence of hotspots located in different modules and the cooperativity of those sited within the same modules. The energetic and structural cooperativity between intramodular residues is also clearly shown in the example of the chymotrypsin inhibitor, where non–binding site residues have a synergistic effect on binding. Interestingly, the binding site of the T cell receptor β chain variable domain 2.1 is contained in one module, which includes structurally distant hot regions displaying positive cooperativity. These findings support the idea that modules possess certain functional and energetic independence. A modular organization of binding sites confers robustness and flexibility to the performance of the functional activity, and facilitates the evolution of protein interactions.

## Author Summary

Proteins are built by domains, which mediate protein–protein interactions involved in different biological activities. A challenging problem in computational biology is the understanding of the domain–domain interaction mechanism. Here, we propose a new approach for the analysis and prediction of domain–domain binding sites. Our computational approach, which relies on the modular division of 3-D domain structures, identifies modular regions involved in binding and can complement previously introduced predictive methods. Further results illustrate that binding sites display a modular configuration. A detailed analysis of protein domains with available experimental binding data revealed that modules are energetically independent from each other, whereas residues within modules contribute cooperatively to the binding energy. The modular composition of binding surfaces may generate high binding affinity and specificity, and facilitate the appearance of new domain binding partners. This advantageous organization of protein structures has been conserved by evolution and may be used to design an effective drug strategy.

## Introduction

Domains constitute the structural and functional units of proteins. They usually mediate protein–protein interactions by binding other domains or smaller peptide motifs. The former are frequently associated with stable interactions, whereas the latter relate to transient interactions [1,2]. It has been previously shown that different organisms use the same domains for domain–domain interactions, emphasizing their evolutionary conservation [3,4]. Important information on protein interactions can be obtained from the domains of interacting proteins. However, mapping domain–domain interactions onto protein–protein networks based on the existing experimental data is not a straightforward task [4,5]. Several groups have proposed statistical approaches based on the integration of multiple biological datasets for inferring domain–domain interactions based on protein–protein interaction networks [4–8]. Although these methods have provided reliable domain–domain interactions, their predictions are limited by the lack and accuracy of data [9]. Identification of domain–domain interaction sites would facilitate the prediction of protein–protein interactions and the understanding of the molecular mechanism of protein function. A number of studies have examined the characteristics of protein–protein interaction sites. Structurally conserved residues at protein–protein interfaces have been found to correlate with experimentally determined hotspots of binding free energy [10,11]. Sequence information has also been used in the identification of hotspots [12]. An early analysis aiming to identify protein–protein binding sites was based on the prediction of surface patches that overlap with interfaces [13]. Sequence conservation and correlated mutations between interacting partners have also been used to identify protein–protein binding sites [14–16]. The combination of sequence and structural information has maximized the predictive power of various methods [17–19]. Nevertheless, new ways of characterizing and predicting binding interfaces are still needed.

Here, we propose a different approach to the analysis of domain–domain binding sites based on the modular decomposition of protein domains [20]. The study was carried out on a large structural dataset of domain–domain interactions based on the protein–protein interaction networks of five different organisms [4]. Our algorithm relies on the representation of domain structures as residue-interacting networks and the modular partitioning of such networks using the edge-betweenness clustering algorithm [21]. Modules, which can be considered as building blocks of domains, are characterized by strong intramodular and weak intermodular residue contacts [20]. Our results revealed that non-overlapping binding sites in a domain, involved in different domain–domain interactions, were mainly located in different modules. These findings support the idea that modular decomposition divides domains into modules, which contain groups of residues displaying a certain specialization for protein binding. Perhaps the most important result in our analysis relates to the fact that a large percentage (72%) of modules that exhibit high modularity values (highly cooperative modules) contain groups of residues belonging to binding sites, suggesting that modularity can be used to identify functional regions. This fact reflects that binding sites contain groups of residues, which act cooperatively for the performance of protein–protein interactions. Although our method relies on single-structure analysis without additional sequence or physico–chemical information, its predictive character is comparable to other protein binding site predictions [22]. Furthermore, the combination of our approach with other characteristics, such as sequence conservation or surface patches, improves the prediction of binding site regions.

Binding sites can be fully contained in one module; however, it is often the case that several modules share a binding site. A detailed inspection of six examples of protein domains (interleukin-4 [IL-4], TEM1-β-lactamase [TEM1], T cell receptor [TCR] β chain variable domain 2.1 [hVβ2.1], growth hormone receptor [GHbp], chymotrypsin inhibitor [CI2], and RNase inhibitor [RI]) illustrates the modular organization of binding sites. Residues within modules generally display energetic cooperativity to protein binding, whereas residues belonging to different modules mainly show energetic additivity. The TEM1–β-lactamase inhibitor protein (BLIP) binding interface modular decomposition clearly illustrates that the energetic contributions of hotspot residues to the complex stability are cooperative within modules and additive between modules [23,24]. The modular division of the CI2-binding site shows the energetic and structural cooperativity existing between intramodular residues, even if they are not involved in the intermolecular interactions [25]. Mutagenesis studies revealed that the hVβ2.1-binding surface contains residues within different hotspot regions separated by more than 20 Å, which are significantly energetically cooperative [26]. Interestingly, these hot regions are contained in one module, reflecting the cooperativity of residues within modules. This example suggests that the modular decomposition of domains, which considers the overall topology of residue-interacting networks rather than local information on interface residue clusters, identifies global cooperative units for protein binding.

Our results suggest that the modular architecture of protein domains confers robustness and flexibility to the performance of the functional activity. The modular configuration of binding interfaces appears to regulate specificity and binding affinity, and suggests how a given domain may bind to different partners. The selective use of different combinations of modules composing a binding site may be an explanation for domain binding promiscuity, and might be an important factor for the evolution of domain–domain interaction networks.

## Results

We previously showed that protein domains consist of modules, which are interconnected by key residues for information transfer between amino acids. These modules can be considered subdomains not only from a structural standpoint, but also in a functional sense. These findings led us to investigate the role of domain modular architecture in the context of protein binding. To this end, we compiled a dataset of 330 protein domains with structurally derived domain–domain interactions based on the protein–protein interaction networks of five different organisms [4] (Table S1). This dataset of domain–domain interactions mediate protein–protein associations involved in a wide variety of cellular processes.

To elucidate how modules characterize binding sites involved in these interactions, we mapped binding sites onto domains and clustered them using a hierarchical agglomerative clustering algorithm (see Materials and Methods). Domain structures were represented as residue-interacting networks [20,27] and decomposed into modules relying on the edge-betweenness clustering algorithm proposed by Newman and Girvan [21,28].

### Modular Separation of Non-Overlapping Binding Sites

**Figure 1:**
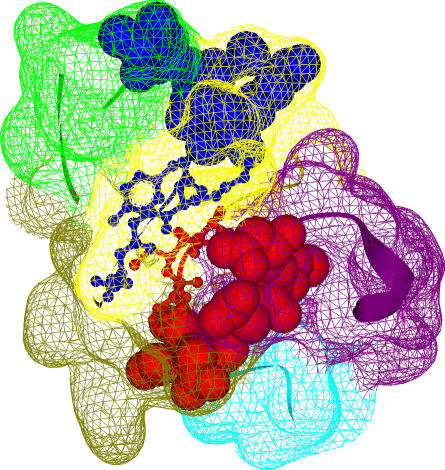
Similarity in Modular Composition and Relative Interface Between Binding Sites The Kringle domain (Pfam ID: PF00051; PDB ID: 1bht) has been chosen as an illustrative example of modular separation of binding sites. The two binding sites A (blue) and B (red) of this domain are represented in spacefill, with their interface residues depicted in balls and sticks. The interface between binding sites A (ten residues) and B (eight residues) involves four and three residues from each binding site, respectively. The relative interface between these binding sites is *C*(*A*,*B*) = 0.39 (see Materials and Methods). The domain has been decomposed into five modules represented by the colored surfaces: 1 (green), 2 (yellow), 3 (olive), 4 (purple), and 5 (cyan). The modular composition of binding sites A and B are (2,8,0,0,0) and (0,2,3,3,0), respectively. The similarity in modular composition of these binding sites is *M*(*A*,*B*) = 0.20.

**Figure 2:**
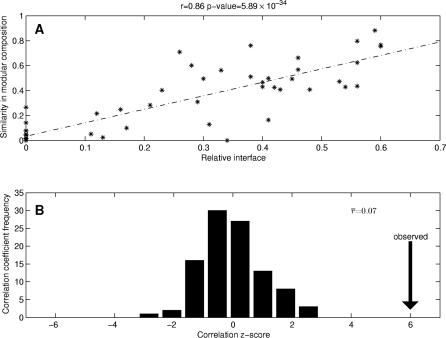
Relationship Between Relative Interface and Similarity in Modular Composition and its Statistical Validation (A) Correlation between relative interface and similarity in modular composition between pairs of domain binding sites. The linear regression line corresponds to the correlation coefficient *r* = 0.86 with a statistically significant *p* = 5.89 × 10^−34^. (B) *Z*-score frequency distribution of the correlation coefficient *r* for all pairs of randomly generated domain binding sites. The correlation coefficients are mainly distributed around 0, illustrating the independence between these two parameters for the random dataset, whereas the correlation for those pairs of domain binding sites in the analyzed dataset (indicated with the vertical arrow) has a statistically significant *z*-score = 6.0.

We aimed to study the domain modular division from a functional standpoint. We addressed the following question: does the modular decomposition lead to the assignment of non-overlapping binding sites to different modules? Initially, we measured the spatial overlap between pairs of binding sites in a domain by using their relative interfaces. Next, we compared the relative interface between binding sites with their modular compositions (see Figure 1 and Materials and Methods). Our results showed that there was a good correlation between the relative interface of each pair of binding sites in a domain and the similarity of their modular compositions. The larger the percentage of contacting residues between two binding sites, the more similar their modular compositions. Conversely, if the interface between two binding sites is small, these binding sites are more likely to be located in different modules (Figure 2A). These findings indicate that the modular division usually assigns non-overlapping binding sites in a domain to distinct modules.

To evaluate the statistical significance of this result, we generated random binding sites in all domains (keeping the same modular decompositions). In this case, there was no correlation between the relative interface between binding sites and the similarity of their modular compositions (Figure 2B). The domain modular partitioning does not tend to allocate randomly generated binding sites into different modules. Thus, modular decomposition divides domains into modules comprising groups of residues exhibiting certain specialization for protein binding.

An illustrative example of a clear modular separation of non-overlapping binding sites is the response regulator receiver domain (Pfam ID: PF00072), which interacts with itself (Protein Data Bank [PDB] ID: 3tmy) [29], and with the sigma-54 interaction domain (Pfam ID: PF00158; PDB ID: 1ny5) [30] through two distinct binding sites located in two different modules (Table S1).

### Modularity and Identification of Binding Site Regions

**Figure 3:**
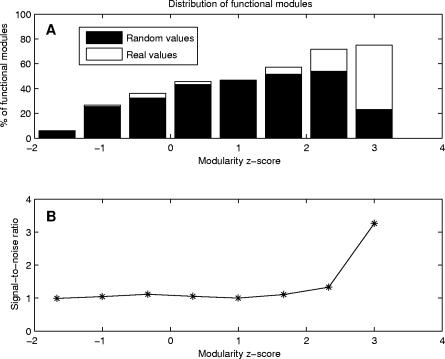
Modularity Distribution of Functional Modules and the Signal-to-Noise Ratio (A) Comparison between modularity distributions for functional modules (including at least 10% of binding site residues) in the analyzed dataset and in the set of randomly generated binding sites. In the analyzed dataset, a large percentage (72%) of modules exhibiting statistically significant values of modularity (*z*-score ≥ 2.0) correspond to functional modules, whereas this tendency is not observed in the random case. (B) Ratio between modularity distributions for the analyzed dataset and the random dataset. The ratio is significantly greater than one where *z*-score values are greater or equal than 2.0.

**Figure 4:**
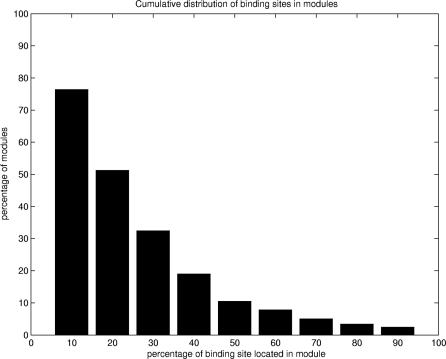
Distribution of Binding Site Residues in Modules Percentages of modules (*y*-axis) containing at least the fraction of binding site residues indicated on the *x*-axis. In the dataset, more than 75% of modules contain at least 10% of binding site residues.

Following the modular partitioning of domains, we sought to identify binding site regions by using an intrinsic characteristic of modules. Modularity compares the percentages of residue contacts within and between modules, measuring the cooperativity of residue interactions in modules (see Materials and Methods). A study based on our dataset of 330 domains indicated that modules with high modularity values generally contain binding site regions. A detailed analysis showed that 72% of all modules exhibiting statistically significant values of modularity (*z*-score ≥ 2.0) contain at least 10% of binding site residues (Figure 3A). Since our goal was to predict binding site regions, rather than all binding site residues, we inspected a significant number of observed modules containing binding site residues. Our results showed that the majority of modules containing binding site residues comprise up to 30% of these residues. Moreover, the cutoff value of 10% was found to be optimal, since it allowed us to analyze a significant number of modules containing binding site residues (Figure 4). Further analysis showed that there was no significant decrease in the accuracy of our method up to a 30% cutoff (see also Figure S1).

**Figure 5:**
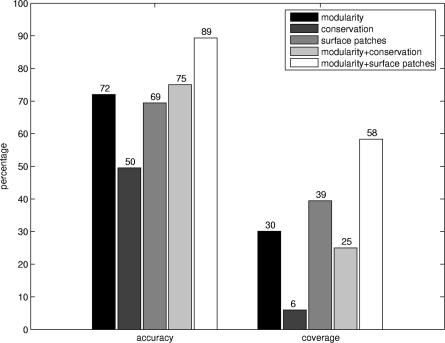
Accuracy and Coverage for Different Methods Accuracy and coverage values calculated for the functionally predicted modules based on modularity, sequence conservation, and surface patches. These values are also represented for the combination of modularity with sequence conservation and surface patches.

A random generation of binding sites for all domains (maintaining the same modular division) proved the significance of our results. High modularity modules do not characterize these randomly generated binding sites (Figure 3A). Furthermore, the distributions of modules containing the annotated and randomly generated binding sites differ significantly in the region of high modularity values (Figure 3B). Our findings indicate that modularity is an informative property that characterizes residue cooperativity in binding site regions. Modularity can be used to complement previously introduced methods for the identification of binding surfaces. Figure 5 compares the predictive performance of our method with the predictions of two other methods—residue conservation and surface patches (see Materials and Methods). Accuracy and coverage values of the modularity and surface patch methods are comparable, whereas they provide greater predictive power than a method based solely on residue conservation (see also Figure S2). Furthermore, combining modularity with sequence conservation or surface patches remarkably improves the predictive performance.

Examples such as Kunitz/bovine pancreatic trypsin inhibitor (Pfam ID: PF00014) and ribosomal protein (Pfam ID: PF00410) domains illustrate our findings. The former interacts with the trypsin domain (Pfam ID: PF00089; PDB ID: 3btw) [31] by using a binding site fully contained in a module with modularity value of 0.172 (*z*-score = 2.23), whereas the latter interacts with itself (PDB ID: 1sei) [32] through a binding site contained in a module with modularity value of 0.176 (*z*-score = 2.32).

### The Modular Architecture of Domain Binding Sites: Examples of Energetic Independence and Cooperativity

Based on our results, we observed that domain-binding sites are frequently divided into several modules (Figure 4). In an attempt to get some insights on the advantages of a modular organization of binding sites, we carried out a detailed analysis of six examples of protein domains with energetic and structural experimental information (IL-4, TEM1, TCR hVβ2.1, GHbp, CI2, and RI).

#### IL-4.

**Figure 6:**
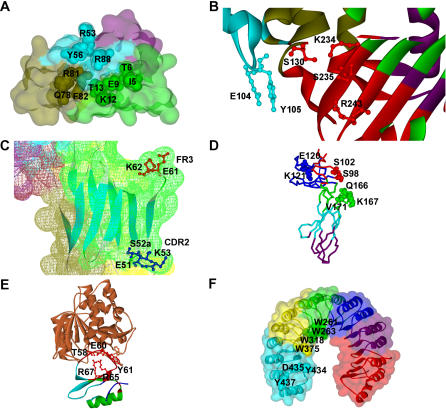
Examples of Modular Configuration of Domain Binding Sites (A) Modular decomposition of the IL-4 domain binding site. The modular decomposition of the IL-4 domain is represented by the colored surface. The binding site of the interaction with its receptor subunit IL-4Rα is configured by three clusters that contribute independently to the binding free energy. The three clusters are respectively located in three different modules. (1) Cluster I is in the green module (I5, T6, E9, K12, T13); (2) cluster II is in the blue module (R53, Y56, R88); and (3) cluster III is in the olive module (Q78, R81, F82). Residues E9 and R88 are the two main hotspots of binding free energy. PDB ID: 2b8u, chain A. (B) Modular decomposition of the TEM1 domain binding site. The ribbon representation is color-coded according to the modular decomposition of the TEM1 domain. The binding site of the interaction with its inhibitor BLIP contains two independent hot regions of binding free energy, which are located in two different modules: (1) red module (S130, K234, S235, R243); and (2) blue module (E104, Y105). PDB ID: 1jtg, chain A. (C) Distant cooperative hot regions within the same module in TCR hVβ2.1. Surface of TCR hVβ2.1 is colored according to its modular decomposition. The two distant cooperative hot regions of binding free energy for the interactions with the superantigen TSST-1 are located in CDR2 (E51, S52a, K53) and FR3 (E61, K62). Both regions are located in the same module (green). PDB ID: 1ktk, chain E. (D) Modular decomposition of hGHbp. Color-coded backbone representation of the modular decomposition of hGHbp. The three clusters in the hydrophilic periphery of the functional epitope, which contribute independently to the binding free energy, are located in three different modules: (1) E120, K121; (2) S98, S102; and (3) Q166, K167, V171. PDB ID: 3hhr, chain B. (E) Modular decomposition of CI2. Representation of the CI2–subtisilin Novo complex. The modular decomposition of CI2 is depicted by color-coded ribbons. Residues R65, R67, T58, E60, and Y61, which display structural and energetic cooperativity, are located within the red module. PDB ID: 2sni, chain I. (F) Modular decomposition of RI. The modular decomposition of RI is represented by the colored surface. Cooperative residues W261, W263, and W318 of the Trp-rich area are contained in the green module, whereas W375, whose contribution to the binding energy is additive with respect to the other tryptophans, belongs to the yellow module. The hotspot region 434–438 is located within the cyan module. PDB ID: 1a4y, chain D.

Human IL-4 is a pleiotropic cytokine that plays a crucial regulatory role in the immune system. IL-4, together with IL-13, elicits various responses in target cells upon binding to a receptor complex consisting of the IL-4Rα and IL-13Rα1 chains. Previous studies have emphasized the modular nature of the IL-4 interaction with its high-affinity receptor subunit IL-4Rα, involving three energetically independent clusters [33]. The high-affinity binding of IL-4 to its receptor is mainly determined by two of these clusters, which contain the hotspots of binding free energy Glu9 and Arg88, respectively [33] (Figure 6A). Interestingly, the modular division of the IL-4 (PDB ID: 2b8u, chain A) illustrates that the three aforementioned clusters are located in three different modules (Figure 6A). Experimental results show that residues belonging to different clusters act independently on the binding free energy. Mutations of amino acids Thr13 (cluster I) and Phe82 (cluster III) do not display cooperativity. In addition, hotspots Glu9 (cluster I) and Arg88 (cluster II) contribute to the binding free energy independently [33]. These two hotspots are used to generate binding affinity and specificity. Thus, in this example we find that modules separate the binding site into regions contributing independently to the binding free energy.

#### TEM1.

TEM1 confers antibiotic resistance to Escherichia coli through enzymatic cleavage of cephalosporins and penicillins. This enzyme is bound and inhibited by BLIP [34]. Experimental results provided by Reichmann et al. [23] indicate that clusters of residues at the TEM1–BLIP interface function as energetically independent binding units. Their analysis leads to the conclusion that interactions are cooperative within clusters and additive between them. Indeed, an extensive mutagenesis study based on two of these clusters shows that in spite of being in structural proximity, they are energetically independent. The modular decomposition of TEM1 (PDB ID: 1jtg, chain A) revealed that these two clusters, which comprise two distinct hotspot regions, are located in different modules (Figure 6B). As in the example of IL-4, the modular organization of the TEM1 binding site illustrates the energetic independence of hotspot regions that may contribute to the evolution of binding affinity and specificity.

#### TCR hV**β**2.1.

Affinity maturation variants of the human TCR hVβ2.1 bind the superantigen toxic shock syndrome toxic 1 (TSST-1) with high affinity [35]. It has been shown that variant residues at positions 51, 52a, 53, and 61, and wild-type residue at position 62, are hotspots of binding free energy for the interaction with TSST-1 [26]. Residues 51, 52a, and 53 form a cluster at the CDR2 loop, whereas residues 61 and 62 are clustered at the end of turn within FR3 (Figure 6C). Experimental results show that amino acids within these two hot regions, which are separated by more than 20 Å, are significantly cooperative. Furthermore, cooperativity between these hot regions is greater than within them [26]. Residues 51 and 53, located at the CDR2 loop, display a level of positive cooperativity with respect to each other, and with residue 61 in the FR3 region. Here, it is clearly illustrated that hotspot regions are not necessarily energetically independent. Interestingly, the modular decomposition of hVβ2.1 (PDB ID: 1ktk, chain E) shows that its TSST-1 binding site is contained within one module (Figure 6C). The analysis of this example suggests that our modular decomposition, which considers the overall topology of domains, rather than local information of their binding sites, can identify structurally distant cooperative regions.

#### hGHbp.

Human growth hormone binds to its cognate receptor to initiate a signaling process, which continues with the recruitment of a second receptor to form the active signaling complex [36]. The extracellular domain of hGHbp contains seven β-strands, organized in a β-sandwich. The hormone-binding site of the receptor contains a central hydrophobic patch of 11 residues (functional epitope), which makes a significant contribution to the binding energy. The functional epitope is surrounded by a hydrophilic periphery, which affects the binding affinity. Mutations of periphery clusters of two to six residues demonstrated that most of clustered mutants improved the binding affinity [37]. Residues within clusters contributed cooperatively to the affinity improvement, whereas combinations of mutated clusters were largely additive [37]. The modular decomposition of the hGHbp (PDB ID: 3hhr, chain B) assigned the three main clusters of the periphery to different modules (Figure 6D), illustrating the cooperativity between residues within modules and the additivity between modules.

#### CI2.

This serine proteinase inhibitor binds very tightly and inhibits subtilisin Novo. CI2 consists of a single domain formed by a four-stranded mixed parallel and antiparallel β-sheet against which an α-helix packs to form a hydrophobic core [38]. This inhibitor docks to the protease via a very rigid extended loop, forming several specific interactions with the active site of the protease. Mutation of hotspot Tyr61 causes significant loss of binding energy, mainly due to loss of packing interaction with subtilisin. Residues Arg65 and Arg67, which are not in contact with subtilisin, provide rigidity to the extended loop by hydrogen bonding and electrostatic interactions with Thr58 and Glu60. Site-directed mutagenesis, including double-mutant cycles, revealed that amino acids Arg65 and Arg67 constitute hotspots of binding free energy, and are energetically coupled [25]. These residues, which are not part of the binding interface, contribute substantially to the binding free energy in an indirect way. The modular decomposition of CI2 (PDB ID: 2sni, chain I) shows that residues Thr58, Glu60, Tyr61, Arg65, and Arg67 are located within one module (Figure 6E). This fact illustrates the structural and energetic cooperativity existing between intramodular residues.

#### RI.

RI binds diverse mammalian RNases with extraordinary high affinity and specificity [39]. RI exhibits a “horseshoe” shape, formed by symmetrical arrangement of 16 homologous tandem units, which facilitates the engulfment of its target. The energetic contribution of different residues of the RI–angiogenin binding interface has been examined using site-directed mutagenesis [40]. The contact region, containing RI 434–438 residues, constitutes a hotspot, with many single-residue replacements producing significant losses of binding energy. Effects of mutations of combinations of hotspot residues proved the existence of a negative cooperativity among these amino acids. Another important region of the binding interface is the Trp-rich area of RI, including Trp261, Trp263, Trp318, and Trp375. Although individual residue mutations in the Trp-rich area cause small or moderate binding energy loss, multiple substitutions are substantially greater than additive. The modular division of the RI (PDB ID: 1a4y, chain D) clearly shows that the hotspot region and Trp-rich area are fully contained in two different modules (Figure 6F). Interestingly, although Trp375 belongs to the Trp-rich area, its contribution to the binding energy is additive with respect to the contribution of the other three tryptophans (3W). The modular decomposition locates Trp375 and 3W in different modules, reflecting their energetic independence (Figure 6F).

## Discussion

Protein domains play a key role in protein–protein interactions. Domains can bind other domains or small peptides by using the same or different binding sites. Here, we propose a new approach to the analysis and identification of domain–domain binding sites, which emphasizes the role of domain modular configuration in domain–domain associations. Domain structures were represented as residue-interacting networks and decomposed into modules by considering their overall topology. The resulting modules exhibit many within-module residue contacts and as few as possible between-module contacts. An extensive study of protein domains revealed that non-overlapping binding sites in a domain, which are involved in different domain–domain interactions, are mainly contained in different modules. This finding shows that domains can be decomposed into modules that comprise groups of residues exhibiting certain specialization for protein binding.

In this study, we used the modularity parameter as a measure of residue cooperativity within a module. Highly cooperative modules, characterized by large modularity values, are composed of residues, which are highly connected among themselves and poorly linked to other modules. Our main result demonstrates that a large percentage (72%) of all modules with high modularity values contain groups of binding site residues, indicating that modularity can be used to predict binding surfaces. Further analysis showed that a combination of modularity and sequence conservation or surface patches improved our predictions. Thus, we suggest that our approach not only complements other methods for predicting domain–domain binding interfaces, but also leads to a deeper understanding of the relationship between protein structure and function.

The analysis of six examples of protein domains disclosed that domain-binding sites often display a modular architecture. Modules are energetically independent from each other, whereas cooperativity is found within each module. Examples, such as IL-4 and TEM1, exemplify the modular configuration of binding sites with distinct hotspot regions located in different modules. Experimental results confirmed the energetic independence of these hotspot regions and the cooperativity of residues within modules. The cooperativity between residues within modules is clearly illustrated with the example of CI2, where non–binding site residues belonging to the same module as binding site residues exert a significant influence on the binding affinity. An interesting example is TCR hVβ2.1, where the modular decomposition unveiled that its binding site, which includes two distant hot regions (more than 20 Å apart), is contained in one module. Mutagenesis studies corroborated a high degree of cooperativity existing between these two distant hot regions. This example illustrates that our approach of modular decomposition considers the overall topology of structures and therefore contains information about cooperativity between groups of structurally distant residues.

To conclude, modules are the basic units of domains, which characterize functional regions. The modular architecture of protein domains provides a deeper insight into the performance of the functional activity, and confers robustness to protein structures against mutational events. Functional specificity and regulation relies on the communication between modules. Highly cooperative regions, whose residues are energetically linked, form domain–domain binding interfaces. The modular composition of binding surfaces may generate high binding affinity and specificity, and facilitate the appearance of new domain binding partners. This advantageous organization of protein structures has been conserved by evolution and may be used to design an effective drug strategy.

## Materials and Methods

### Dataset.

We compiled a dataset of 330 protein domains involving 370 domain–domain interactions from the database provided by Itzhaki et al. [4] This database was obtained by mapping structurally derived domain–domain interactions onto the cellular protein–protein interaction network of five different organisms (Escherichia coli, Saccharomyces cerevisiae, Caenorhabditis elegans, Drosophila melanogaster, and Homo sapiens)*.* Our initial dataset contained all single-chain domains with representative structures of domain–domain interactions in the iPfam database [41]. Using multiple sequence alignments provided by iPfam, we mapped all the binding sites of each domain onto its representative structure (Table S1). We selected only binding sites containing at least 80% of their residues within the representative domain structures. All structure images were created using DS ViewerPro 6.0 [42].

### Network analysis of domain structures.

Residues *i* and *j* were considered to be in contact if at least one atom corresponding to residue *i* was at a distance of less than or equal to 5 Å from an atom from residue *j*. This value approximates the upper limit for attractive London–van der Waals forces [43]. We modeled the PDB structures of the representative domains as graphs, with residues corresponding to vertices, and their contacts to edges. These networks were subsequently decomposed into modules using the edge-betweenness clustering algorithm proposed by Girvan and Newman [21,28], based on the iterative removal of edges with the highest number of paths running through it (see also Figure S3). We used the parallel implementation PEBC (parallel edge-betweenness clustering) [44] of the algorithm.

We used the previously introduced expression for the modularity of each module *m* [20]:

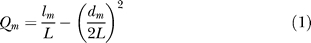
where *L* is the number of edges in the network, *l_m_* is the number of edges between nodes in module *m*, and *d_m_* is the sum of the degrees of nodes in module *m*. Modules with higher *Q_m_* contain many within-module edges, whereas random partitions of the network have an expected value of *Q_m_* = 0.

### Binding site analysis.

*Binding site clustering.* In order to detect whether a domain is interacting with different domains using non-overlapping binding sites, we clustered the list of binding sites corresponding to each domain in the dataset. First, we defined a distance matrix for all pairs of binding sites as:

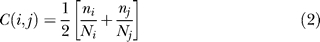
where *n_i_* and *n_j_* are the number of residues in binding sites *i* and *j* that have contacts with the other binding sites *j* and *i*, respectively. *N_i_* and *N_j_* are the total number of residues belonging to each binding site. Two binding sites *i* and *j* were considered as non-overlapping if *C*(*i*, *j*) < 0.7.

Our clustering protocol was based on the hierarchical agglomerative clustering algorithm (see also Figure S4), defined as follows: (1) find the closest pair of binding sites in the distance matrix; (2) merge these two binding sites into a new single binding site if the distance between them is *C*(*i*, *j*) < 0.7; and (3) compute the distance matrix for the new reduced list of binding sites. The clustering process terminates when the distances between all pairs of binding sites are above the threshold, obtaining a set of mutually non-overlapping binding sites in the domain.

*Relative interface between binding sites.* We defined the relative interface between two binding sites as in Equation 2. This parameter represents the averaged proportion of binding site contacting residues, and is a measure of closeness between these binding sites. *C*(*i*, *j*) varies from 0 to 1. Values close to 0 imply a small relative interface, indicating a clear structural separation between both binding sites, whereas values close to 1 appear when almost all residues in both binding sites are on the interface, illustrating their proximity.

*Similarity of binding site modular compositions.* We defined for each binding site *j* a vector **m** *_j_* representing its modular composition as follows:

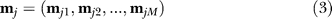
where **m** *_jk_* is the number of residues of binding site *j* in module *k*; and *M* is the total number of modules in which the domain has been decomposed.

The modular composition similarity between two binding sites *i* and *j* is defined as the uncentered Pearson correlation coefficient between their respective vectors of modular composition:

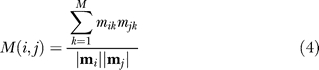
where 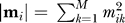 , and 
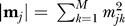
 are the Euclidean norms of vector *i* and *j*, respectively.

*M*(*i*, *j*) varies from 0 to 1. Values close to 0 show significant differences in the modular compositions of each binding site, whereas values close to 1 correspond to binding sites with almost identical modular compositions.

### Evaluation of performance.

*Random generation of binding sites.* To test the statistical significance of our studies, we generated a list of random binding sites for each domain, keeping the same number and size of the original binding sites. The random binding sites were generated in the following way: (1) we randomly selected one of the residues in each binding site as the seed residue for the new binding site; and (2) we iteratively added more random neighbors to the new binding site until the number of residues on it equaled the size of the original binding site. In the case of domains with more than one binding site, we checked that all pairs of binding sites in the corresponding list verified *C*(*i*, *j*) < 0.7; otherwise, the random generation of binding sites for this domain was repeated until such condition was reached. We generated 500 random realizations for each binding site of each domain of our dataset.

*Accuracy and coverage.* The accuracy and coverage for the prediction methods were defined as:

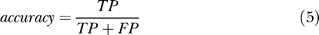


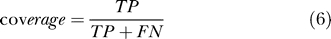
where *TP*, *FP*, and *FN* are the number of true positives, false positives, and false negatives, respectively.

*Conservation analysis.* Residue conservation scores were determined for each representative domain structure from the ConSurf-HSSP database [45]. A residue was considered as conserved if its score was greater or equal to 9.

*Patch analysis.* Predictions of surface patches for the representative domain structures were determined from the SHARP^2^ server [46]. We considered the best three predicted overlapping patches.

## Supporting Information

Figure S1 Accuracy Values for Different Percentages of Binding Site Residues within a Module Accuracy values for different ratios of binding site residues located in the functional modules. Accuracy is around 72% for low-detection values under 20% of binding site residues.
(2.4 MB TIF)

Figure S2 Sequence Conservation Distribution for Functional Modules Sequence conservation distribution for functional modules (including at least 10% of binding site residues) in the analyzed set. There is no clear tendency for functional modules to exhibit statistically significant values of sequence conservation (*z*-score ≥ 2.0).
(2.5 MB TIF)

Figure S3 Edge-Betweenness Clustering Algorithm The modular partition of the residue interacting network of domain structures is based on the edge-betweenness clustering algorithm, which is illustrated.
(1) Initially in (A), the betweenness is computed for all edges in the network (number of shortest paths between pairs of vertices that run along it). The edge with the highest betweenness is depicted in red.
(2) In (B), the edge with the highest betweenness is removed.
(3) Next, recalculate betweennesses for the remaining edges.
(4) Repeat (2) until no edges remain.
As shown (C) and (D), the network has been partitioned into two modules. In (E), the network has been partitioned into three modules. The optimal partition algorithm stops when the maximum value of the network modularity is reached.
(6.8 MB TIF)

Figure S4 Clustering of the Set of Binding Sites for Each Domain In this example, a domain interacts with five different domains using binding sites B1 to B5. However, pairs of binding sites (B1, B2), and (B3, B4), have significant numbers of residues in contact, and therefore their relative interfaces are *C*(*i*, *j*) < 0.7. After the clustering procedure, (B1, B2) and (B3, B4) are merged into binding sites B1^*^ and B2^*^, respectively, while B5 is assigned to B3^*^, obtaining a set of three mutually non-overlapping binding sites in the domain.
(6.8 MB TIF)

Table S1 List of Domain–Domain Interactions This table contains the Pfam ID codes corresponding to the 370 domain–domain interactions compiled for this study. The PDB ID code of the structure used as template for each domain is also given.
(77 KB XLS)

## Abbreviations

BLIP: β-lactamase inhibitor protein
CI2: chymotrypsin inhibitor
GHbp: growth hormone receptor
hVβ2.1: human β chain variable domain 2.1
IL-4: interleukin-4
RI: RNase inhibitor
TCR: T cell receptor
TEM1: TEM1-β-lactamase
TSST-1: toxic shock syndrome toxic 1
